# Effect Of α2-Adrenergic Agonists And Antagonists On Cytokine Release From Human Lung Macrophages Cultured In Vitro

**Published:** 2016-11-01

**Authors:** O. Piazza, R.I. Staiano, E. De Robertis, G. Conti, V. Di Crescenzo, S. Loffredo, G. Marone, G. Zito Marinosci, M. M. Cataldi

**Affiliations:** 1 Università di Salerno, Department of Medicine and Surgery, Via Allende, 84081 Baronissi, (SA) Italy; 2 Università degli Studi di Napoli Federico II, Department of Translational Medical Sciences and Center for Basic and Clinical Immunology Research (CISI), Naples, Italy; 3 Università degli Studi di Napoli Federico II, Department of Neurosciences, Naples, Italy; 4 Federico II University Hospital, Naples, Italy; 5 Università Cattolica del Sacro Cuore, Anaesthesiology and Intensive Care, Rome, Italy

**Keywords:** clonidine, dexmedetomidine, medetomidine, yohimbine, cytokines, macrophages

## Abstract

The most trusted hypothesis to explain how α2-adrenergic agonists may preserve pulmonary functions in critically ill patients is that they directly act on macrophages by interfering with an autocrine/paracrine adrenergic system that controls cytokine release through locally synthetized noradrenaline and α1- and α2-adrenoreceptors. We tested this hypothesis in primary cultures of resident macrophages from human lung (HLMs). HLMs were isolated by centrifugation on percoll gradients from macroscopically healthy human lung tissue obtained from four different patients at the time of lung resection for cancer. HLMs from these patients showed a significant expression of α2A, α2B and α2C adrenoreceptors both at the mRNA and at the protein level. To evaluate whether α2 adrenoreceptors controlled cytokine release from HMLs, we measured IL-6, IL-8 and TNF-α concentrations in the culture medium in basal conditions and after preincubation with several α2-adrenergic agonists or antagonists. Neither the pretreatment with the α2-adrenergic agonists clonidine, medetomidine or dexdemetomidine or with the α2-adrenergic antagonist yohimbine caused significant changes in the response of any of these cytokines to LPS. These results show that, different from what reported in rodents, clonidine and dexdemetomidine do not directly suppress cytokine release from human pulmonary macrophages. This suggests that alternative mechanisms such as effects on immune cells activation or the modulation of autonomic neurotransmission could be responsible for the beneficial effects of these drugs on lung function in critical patients.

## I. INTRODUCTION

α2-adrenergic agonists including dexmedetomidine and clonidine exert sedative, analgesic, and anxiolytic effects by acting at different levels. More specifically, they decrease noradrenaline release in locus coeruleus hence activating descending inhibitory pathways that reach the posterior horns of the spinal cord to inhibit the transmission of painful stimuli to the brain (1). α_2_-adrenergic receptors are also expressed in projecting neurons of the dorsal horn and inhibit their activity. In addition, a possible direct blocking effect of α_2_-adrenergic agonists on Aδ and C fibres has been described and could account for the ability of these drugs to enhance the peripheral nerve blocking activity of local anaesthetics (2). Finally, α_2_-adrenergic agonists exert central sedative effects acting at the level of thalamic nuclei. By this mechanism they also synergize with opioids hence lowering the need for opiate medication during post-operative pain. Since they can induce a good level of sedation with less respiratory and cardiovascular depression than conventional centrally-acting sedative drugs, α_2_-adrenergic agonists are used very often in the intensive care unit (ICU). Their sedative effects may be helpful, indeed, in seriously ill patients such as those with sepsis or respiratory distress syndrome. Clonidine is usually preferred in the pediatric patients whereas dexmedetomidine is the α_2_-adrenergic agonist more commonly used in adults (3). Although many side effects have been described for these drugs such as hypotension, rebound tachycardia and hypertension after withdrawal, a moderate, not clinically relevant bradycardia is the only one that is frequently observed in critical patients (4).

An important argument supporting the use of α_2_-adrenergic drugs in the ICU is the clinical evidence that they may favourably impact on the clinical course in critical patients. For instance, it has been shown that the length of stay in the ICU and time to extubation are significantly shorter in critically ill patients that receive dexmedetomidine instead of propofol or benzodiazepines (5). In addition, a systematic review of literature (6) reported that dexmedetomidine improves short-term mortality in sepsis patients, compared with other sedatives, even though this conclusion needs further studies to be confirmed.

It is currently unknown by which mechanism dexmedetomidine could exert these positive effects on morbidity and mortality of critical patients. Among the proposed explanations it has been suggested that it could have significant antinflammatory effects that could be useful, for instance, in maintaining alveolar gas exchanges in the inflamed lung. Several preclinical studies seem to support this hypothesis. For instance, a decrease in cytokine release in response to LPS has been observed upon incubation with dexmedetomidine in the macrophagic cell line RAW264.7 (7) and in cultured astrocytes (8) and microglia (9). Moreover, Xu et al (10) showed that in a mouse model of acute lung injury, the pretreatment with dexmedetomidine significantly blunted the increase in plasma TNF-α the by a mechanism dependent on the inhibition of the MAPK signalling pathway. Few studies investigated the anti-inflammatory effects of dexmedetomidine in humans and most of them were based on indirect evidence. For instance, in 20 patients with sepsis, Memis et al showed that TNF-α, IL-1β and IL-6 levels in plasma were significantly lower after 24 hours of sedation with dexedemetomidine in comparison with controls that did not receive this drug (11). Gao et al (12) compared two groups of patients undergoing one-lung ventilation for lung cancer surgery, the first receiving dexmedetomidine intraoperatively and the latter not. They showed that the peripheral concentrations of TNF-α were significantly lower in patients treated with the α_2_-adrenergic agonist. Moreover, in this group of patients the levels of the heme oxygenase mRNA in the removed lung tissue was higher than in the other group. Considering that heme oxygenase has antinflammatory effects this finding stands for a direct antinflammatory effect of dexmedetomidine on the lung. Up to date no study investigated yet whether the possible antinflammatory effects of dexmedetomidine on the lung are directly exerted at the level of inflammatory cells such as lung monocytes although the evidence that this drug lowers proinflammatory mediator production in whole human blood in vitro stands for a direct effect on inflammatory cells (13). Therefore, we designed the present study to investigate whether primary macrophages isolated from human lung do express α2-adrenergic receptors and respond to α_2_-adrenergic agonists by decreasing cytokine release.

## II. METHODOLOGY

This study was performed on macroscopically normal tissue obtained from four patients. No patient was allergic, asthmatic or affected by bronchitis. The study protocol was approved by the ethics authority and all patients gave their written informed consent to take part to this study.

### 2.1. HLM isolation, purification and culture

Human macrophages were purified as described elsewhere (14). Lung tissue was mechanically dispersed and the macrophage suspension was enriched (75–85%) by flotation over Percoll® density gradients. After being suspended in RPMI-1640 containing 5% FCS, 2mM Lglutamine, and 1% antibiotic-antimycotic solution at the final density of 2×10^6^ cells/mL, cells were allowed to adhere to plastic dishes at 37°C in a 5% CO_2_ atmosphere. After 12 h, the medium was removed and the plates were gently washed with RPMI-1640. More than 98% of adherent cells were macrophages, as assessed by flow cytometry analysis and a-naphthylacetate esterase staining.

### 2.2. RT-PCR for ADRA2A, ADRA2B and ADRA2C

Total RNA from HLMs and from breast adenocarcinoma MCF7 cells, that were used as a positive control because constitutively expressing all the three ADR2A isoforms (15) was extracted using the SV 96 total RNA isolation system (Promega, Madison, WI) and treated with RNase-free DNase I. Reverse transcription was performed as previously described (16). ADRA2A, ADRA2B and ADRA2C mRNA expression was evaluated by semiquantitative PCR using specific primers that were designed with Beacon Designer 3.0 software (Biorad Laboratories, Milan, Italy) according to the corresponding cDNA sequences published in GenBank accession No. NM_000681.3, Homo sapiens adrenoreceptor alpha 2 (ADRA2A); NM_000682.5 ADRA2B; NM_000683.3 ADRA2C) and that were synthesized by Invitrogen (Carlsbad, CA). Specifically, we used the following primers: ADRA2A forward: 5′-ACTGGACTACAAGGGCATGG -3′, reverse: 5′-ACATCAAAACCAAGGCCAAG -3′; ADRA2B forward: 5′-CCTGTTTTCGGATCTGTGGT -3′, reverse: 5′-CTGCAAAGCCTTTCATCTCC -3′; ADRA2C forward: 5′-CCGGTCATCTACACGGTCTT -3′, reverse: 5′-ATCTCTCTGCCAAGCTCCTG -3′. Similar amounts of cDNAs from HLMs and MCF-7 were amplified according the following PCR protocol: denaturation at 95°C for 10 minutes, amplification with 35 cycles of 20 second, denaturation (94°C) and 30 second annealing (55°C). The PCR products were separated on 2% agarose gel, stained with ethidium bromide and visualized under the image analysis system Chemidoc XRS (Biorad).

### 2.3. Western blot analysis of α_2A_-AR, α_2B_-AR e α_2C_-AR

Total proteins were extracted from HLMs and MCF-7 cells using the following lysis buffer: 20 mM Tris pH 7.5, 5 mM EDTA, 1 mM PMSF, 2 mM benzamidine, 10 μg/ml leupeptin, 10 mM NaF, 150 mM NaCl, 1% Nonidet P-40 and 5% glycerol (17).

Cell lysates were kept on ice for 20 minutes then microfuged (14,000 rpm, 4°C, 20 min). An aliquot of protein extracts from cell lysates was drawn and the protein content was measured with the BCA Protein Assay Kit (Novagen, Merck Biosciences, San Diego, CA). The remaining protein extracts were diluted in lithium dodecyl-sulfate sample buffer with 2.5% 2-mercaptoethanol, and boiled before storage at −80°C. Equal protein extracts (30–50 μg per sample) were separated on 4–12% Bis-Tris gels (NuPAGE, Novex, Invitrogen) and transferred to a nitrocellulose membrane (Schleicher & Schuell, Dassel, Germany) together with a biotinylated protein ladder (Cell Signaling, Beverly, MA). After immersion overnight in TBST (50 mM Tris pH 7.5, 150 mM NaCl and 0.05% Tween 20) containing 5% nonfat dry milk (Biorad), membranes were washed three times (10 min each) with TBST then incubated (4°C, overnight) with goat anti-α_2A_-adrenoreceptor (1:500), goat anti- α_2B_- adrenoreceptor (1:500), or rabbit anti- α_2C_-adrenoreceptor (1:1000). The membranes were washed then incubated (22°C, 1 hour) with HRP-conjugated rabbit anti-goat IgG Ab or donkey anti-rabbit IgG Ab together with HRP-conjugated anti-biotin Ab. Membrane-bound antibodies were visualized with the ECL Plus Western blotting detection system (GE Healthcare) under the Chemidoc XRS.

### 2.4. Pharmacological treatments and Cytokine assay

TNF-α, IL-8 and IL-6 were measured in the culture medium of HLM cells stimulated with LPS (100 ng/ml for 6 hours) after a 30 min preincubation either with vehicle or with metedomidine or dexmedetomidine (0.1–10 μM), clonidine (0,3–3 μM) or yohimbine (1–10 μM). At the end of the incubation with LPS, cell culture medium was collected and centrifuged at 1,000 g for 5 min. Then the supernatant was collected and stored at −80°C till the assay. To assess whether the pharmacological treatments affected cell viability, the trypan blue exclusion test was performed at the end of all the experiments and it always showed that more than 95% of the plated cells were still alive. Cytokine assays were all performed in duplicate using commercially available ELISA kits from R&D System, (Minnesota, USA). The results were expressed in nanograms per ml of supernatant fluids.

### 2.5. Statistical analysis

All data were expressed as mean ± SEM. Statistical analysis was performed with one-way analysis of variance (ANOVA) followed by Bonferroni post-hoc test using the *Analyse-it 2.16* statistics package for Microsoft Excel (Analyse-it Software, Ltd., Leeds, United Kingdom). Differences were considered statistically significant when p< 0.05.

## III. RESULTS

**Figure 1:**
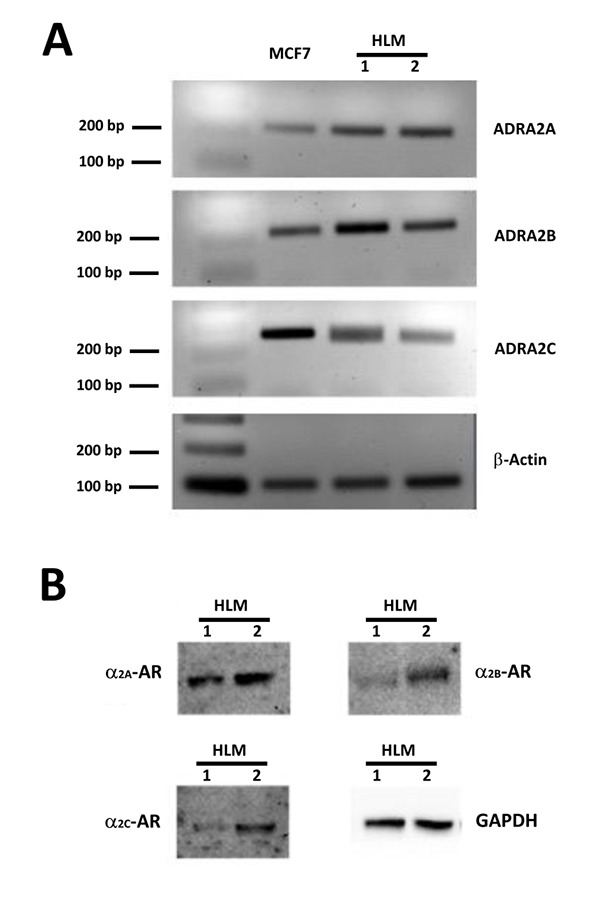
ADRA2A, ADRA2B, ADRA2C expression in HLMs. *Panel A*: RT-PCR performed with primers specific for ADRA2A, ADRA2B and ADRA2C. Gels were loaded with retrotranscripts from lung tissues of two different patients (Lines 1 and 2) and from MCF-7 cells, a breast cancer cell known to express all the three α_2_-adrenoreceptor isoforms that was used as an internal reference. In all the PCR reactions bands of about 200 bp were amplified, a size corresponding to what expected for ADRA2A (211 bp; first line), ADRA2B (230 bp; second line) and ADRA2C (242 bp; third line). β actin was used as housekeeping gene product or normalization. *Panel B*: Western blotting performed using anti-α2A-, anti α2B- and anti- α2C-adrenoreceptor antibodies and cellular lysates from HLMs obtained from two different patients preparations. GAPDH immunoreactivity was used for normalization. In both HLM preparations, immunoreactive bands of the expected size were obtained for all the three α2-adrenoreceptor isoforms (70, 62 and 60 kDa for α2A-, α2B- and α2C-adrenoreceptors, respectively).

To establish whether human lung macrophages do express α_2_-adrenergic receptors we performed PCR and Western blot experiments on HMLs purified from the pulmonary tissue of four patients undergoing surgery for lung cancer. As detailed in the methods section, only macroscopically healthy portions of resected lung not infiltrated by cancer were used for HLM preparation. As shown in Fig. 1A, PCR amplified DNA fragments of the expected size for ADRA2A (211 bp), ADRA2B (230 bp) and ADRA2C (242 bp) indicating that all these three adrenergic receptor isoforms are expressed in HLMs. Western blot experiments showed a significant expression of all the three α_2_-adrenergic receptor isoforms also at the protein level (Fig. 1B).

**Figure 2:**
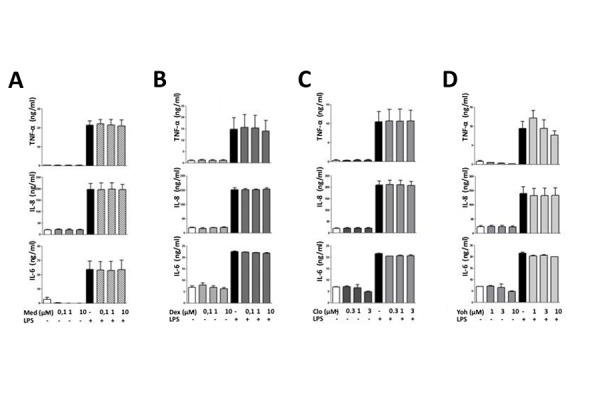
Effect of α_2_-adrenergic agonists and antagonists on in vitro cytokine release from HMLs. The bar graphs show the effect of vehicle and of progressively higher concentrations of the α_2_-adrenergic agonists dexmedetomidine (0.1–10 μM) (panel A), medetomidine (0.1–10 μM) (panel B) and clonidine (0,3–3 μM) (panel C) and of the α_2_-adrenergic antagonist yohimbine (1–10 μM) (panel D) on LPS-induced TNF-α, IL-8 and IL-6 release in the culture medium from HLMs. HLMs were preincubated with the aforementioned drugs for 30 min before adding LPS (100 ng/ml) to induce TNF-α, IL-8 and IL-6 release. The concentrations of these cytokines in culture medium were measured by ELISA 18 hours after LPS addition. Each bar represents as the mean± SEM of the concentrations of the respective cytokine obtained in four different experimental sessions.

To assess whether α_2_-adrenergic drugs could directly affect macrophage activity, we evaluated the effect of the α_2_-adrenergic agonist yohimbine, and of the antagonists, clonidine, medetomidine, and dexdemetomidine on LPS-induced cytokine release from cultured HLMs. When HLMs were incubated with LPS for 18 hours in the absence of these drugs, the concentrations of TNF-α, IL-6 and IL-8 in culture medium increased six-, three- and eight-fold, respectively (TNF-α, from 0.71±0.37 to 11.52±2.85 ng/ml, IL-6 from 6.93±0.09 to 21.84±0.54 ng/ml; IL-8 from 20.08±3.09 to 166.87±27.77 ng/ml; p ≤ 0.05 for all these cytokines). As shown in Fig. 2, no significant difference in the LPS-induced stimulation of the release of any of these cytokines was observed when HML cells were preincubated for 30 min with yohimbine, clonidine, medetomidine, or dexmedetomidine.

## IV. DISCUSSION

In this study, we evaluated the effect on human lung macrophages of several drugs acting on α-adrenergic receptors. The main result that we obtained was that although HLM do express multiple isoforms of α_2_-adrenergic receptors neither the pharmacological stimulation or the inhibition of these receptors does affect cytokine release by these cells.

The main reason that prompted us to investigate the issue of the adrenergic regulation of lung macrophages was the clinical evidence that the α_2_-adrenergic agonist dexmedetomidine reduces inflammation and injury in critically ill humans (5) and in several experimental models of lung damage including sepsis (18), liver transplantation (19), ischemia-reperfusion (20, 21) ventilator-induced lung injury (22) or hemorrhagic shock (23). Although the mechanism responsible for this clinically relevant effect remains obscure, it has been repeatedly suggested that it could relay on the ability of α_2_-adrenergic drugs to inhibit the activity of macrophagic cells hence preventing lung inflammation, which is a major contributor of respiratory impairment in critically ill patients. This hypothesis, however, is mainly supported by data obtained in experimental systems that are significantly different from a failing lung. Starting from the seminal observations of Spengler et al (24), it has been repeatedly shown that α_2_ adrenoreceptors control cytokine release in primary macrophagic cell cultures (25) and in continuous macrophagic cell lines such as RAW264.7, cells in which 1μM dexmedetomidine inhibited LPS-induced IL-1β, TNF-α, IL-6, and IL-10 production (7) and decreased the release of proinflammatory High Mobility Group Box 1 (HMGB1) proteins (26). Direct anti-inflammatory effects of dexmedetomidine have also been documented in whole blood (13), in primary cultures of rat astrocytes (8) or microglia (27). Conversely, very few data are available on the effect exerted by dexmedetomidine on lung macrophages. Jiang et al (28) showed that this α_2_-adrenergic agonist prevents H_2_O_2_-induced oxidative cell damage in NR8383 cells, a continuous cell line derived from lung macrophages. Moreover, dexmedetomidine significantly lowers the concentration of cytokines in bronchoalveolar lavage fluid of rats with lung ischemia-reperfusion injury (21) or sepsis (29). Our study is the first to directly investigate the effect of dexmedetomidine on human pulmonary macrophages. A wealth of data has been accumulated supporting the idea that resident macrophages differ from one tissue to the other (30–32). Therefore, the choice of the source of macrophages on which pharmacologically active substances should be tested could be not a trivial issue. In this perspective, our study adds new information on what is already known on the effect of dexmedetomidine and other drugs acting on α_2_-adrenergic receptors in the human lung.

Our finding that drugs acting on α_2_-adrenergic receptors do not affect cytokine release from HLMs is in contrast with current evidence of an anti-inflammatory role of α_2_-adrenergic agonists in a plethora of serious lung diseases, also in humans. This raises the question of how these anti-inflammatory effects could be exerted independently from a direct action on HLMs. A first point that should be considered is that, although it has been clearly demonstrated that both macrophages and polymorphonucleates produce and release their own cathecholamines that can autocrinally and paracrinally act on these cells (33), a clear demonstration that this is the main mechanism responsible for the adrenergic regulation of lung inflammation in humans has never been provided. Conversely, a much more relevant source of cathecolamines, especially in the setting of a critically ill patient, could be represented by the massive activation of the orthosympathetic system that has been shown to have an important role both in the formation of pulmonary edema and in lung inflammation (34). In this perspective, testing the effect of α_2_-adrenergic drugs on isolated macrophages *in vitro* could not reliably reproduce what happens in the lung *in vivo* when cathecolamines are released from adrenergic terminals and strongly stimulate not only α_2_- but also α_1_-adrenoreceptors on macrophages. It has been suggested, indeed, that cathecolamines stimulate cytokine release from inflammatory cells by a α_1_-adrenoreceptor dependent mechanism that is negatively modulated by α2-adrenoreceptors (35). This implies that when the effect of α_2_-adrenergic drugs was tested on isolated macrophages these compounds could have been ineffective simply because the α1-system that they are supposed to modulate was not stimulated at all. It also has to be considered that part of the effects of adrenergic drugs on pulmonary inflammation could be indirect and be exerted at the level of adrenergic modulation of cholinergic system. It has been reported, indeed, that macrophages do express nicotinic receptors whose stimulation suppress cytokine synthesis by inhibiting the translocation of NF-κB from the cytoplasm to the nucleus (36) and Liu et al recently (37) showed that in a model of sepsis-induced lung damage in the rat, the antinflammatory effect of dexmedetomidine is attenuated by the nicotinic antagonist α-bungarotoxin. This suggests that α_2_-adrenergic agonists could reduce lung inflammation by enhancing the activity of the parasympathetic system and consequently the local release of acethylcholine. An additional hypothesis that could explain why α_2_-adrenergic drugs were ineffective in our experimental system is that that the main effect of these drugs could be exerted not on resident macrophages but on inflammatory cells that penetrate into the lung in the presence of a strong inflammatory stimulus such as monocytes or polymorphonucleates. For instance, α_2_-adrenergic drugs could reduce lung inflammation by impairing chemotaxis. Previous evidence has been reported, indeed, that α_2_-adrenergic receptors control phagocytosis and chemotaxis in primary cultures of rat peritoneal macrophages (38) and exert a modulatory role on pleural neutrophilia elicited by the evoked by the instillation of LPS in the pleural cavity in the rat (39). Our finding that dexedemetomidine and clonidine do not directly suppress cytokine release from resident lung macrophages could have interesting clinical implications. It suggests, indeed, that these drugs do not inhibit the basal “immunological surveillance” activity of these cells whereas they could impair by any of the aforementioned proposed mechanisms the supramaximal macrophagic activation that takes place in the presence of serious lung damage such as in sepsis or in ventilator-induced lung injury (40). If this conclusion would be confirmed this could represent an additional important argument in support of the use of α_2_-adrenergic agonists in critically ill patients. It would provide indeed a rationale to exclude that these drugs could depress local lung defenses, which could be extremely dangerous in the ICU.

## V. CONCLUSION

In conclusion, we demonstrated that the documented ability of dexdemetomidine and clonidine to reduce lung inflammation in critically ill patients is not dependent on a direct suppression of the activity of resident lung macrophages. While further studies will be necessary to clarify the mechanism responsible for the antinflammatory effect of these drugs, our data suggest that it does not directly impair the “immunological surveillance” activity of these cells.
